# MSD-Net: Multi-Scale Discriminative Network for COVID-19 Lung Infection Segmentation on CT

**DOI:** 10.1109/ACCESS.2020.3027738

**Published:** 2020-09-29

**Authors:** Bingbing Zheng, Yaoqi Liu, Yu Zhu, Fuli Yu, Tianjiao Jiang, Dawei Yang, Tao Xu

**Affiliations:** 1 School of Information Science and EngineeringEast China University of Science and Technology 47860 Shanghai 200237 China; 2 The Affiliated Hospital of Qingdao University Qingdao 266000 China; 3 Department of Pulmonary MedicineZhongshan HospitalFudan University 12478 Shanghai 200032 China

**Keywords:** COVID-19, CT, deep learning, MSD segmentation network

## Abstract

Since the first patient reported in December 2019, 2019 novel coronavirus disease (COVID-19) has become global pandemic with more than 10 million total confirmed cases and 500 thousand related deaths. Using deep learning methods to quickly identify COVID-19 and accurately segment the infected area can help control the outbreak and assist in treatment. Computed tomography (CT) as a fast and easy clinical method, it is suitable for assisting in diagnosis and treatment of COVID-19. According to clinical manifestations, COVID-19 lung infection areas can be divided into three categories: ground-glass opacities, interstitial infiltrates and consolidation. We proposed a multi-scale discriminative network (MSD-Net) for multi-class segmentation of COVID-19 lung infection on CT. In the MSD-Net, we proposed pyramid convolution block (PCB), channel attention block (CAB) and residual refinement block (RRB). The PCB can increase the receptive field by using different numbers and different sizes of kernels, which strengthened the ability to segment the infected areas of different sizes. The CAB was used to fusion the input of the two stages and focus features on the area to be segmented. The role of RRB was to refine the feature maps. Experimental results showed that the dice similarity coefficient (DSC) of the three infection categories were 0.7422,0.7384,0.8769 respectively. For sensitivity and specificity, the results of three infection categories were (0.8593, 0.9742), (0.8268,0.9869) and (0.8645,0.9889) respectively. The experimental results demonstrated that the network proposed in this paper can effectively segment the COVID-19 infection on CT images. It can be adopted for assisting in diagnosis and treatment of COVID-19.

## I. Introduction

Since December 2019, some hospitals in Wuhan City, Hubei Province had found multiple cases of unexplained pneumonia with a history of exposure to the seafood market in South China. It has now been confirmed as an acute respiratory infection caused by 2019 novel coronavirus (2019-nCoV) [1]–[3]. The pneumonia caused by 2019-nCoV is named ’Corona Virus Disease 2019’ (COVID-19) by World Health Organization [4]. Up to June 30th, COVID-19 has become global pandemic with more than 10 million total confirmed cases and 500 thousand related deaths [5]. The number of infections and related deaths is still rising fast every day in the world.

Early identification of patients, quarantine and appropriate treatment are best approaches to slow and stop its rapid spread. The SARS-CoV-2 real-time reverse transcription polymerase chain reaction (RT-PCR) test of upper respiratory tract specimen is most recommended for suspected ones according to WHO clinical management [6]. Nevertheless, RNA test can identify whether a patient is infected with COVID-19, but it does not identify the infection degree of the patient. Therefore, it is difficult to carry out targeted treatment. Computed tomography (CT) provides a non-invasive and effective method for detecting the manifestations of viral pneumonia. Computed Tomography can help identify whether the patient is infected with COVID-19, and display the evolution of the lung infection area of the patient at different periods. It may assist doctors in targeted treatment, as well as study the infection process of COVID-19. Computed Tomography is a key component of the diagnostic procedure for suspected patients and its CT manifestations have been emphasized in several recent reports [7], [8]. The segmentation of infected lesions by CT scan is important for the quantitative measurement of disease progression [9].

Typical COVID-19 presentation includes ground glass opacity mainly distributed in bilateral lower lobes, peripheral area under the pleura for mild cases, whereas consolidation and mixed ones are more common in severe cases, as well as thickened interlobular septa, involvement of the center areas, pleural effusion and enlarged mediastinal nodes [10]. According to [11], pulmonary CT infection areas of COVID-19 patients can be divided into three categories: ground-glass opacities, interstitial infiltrates and consolidation.

**FIGURE 1.**
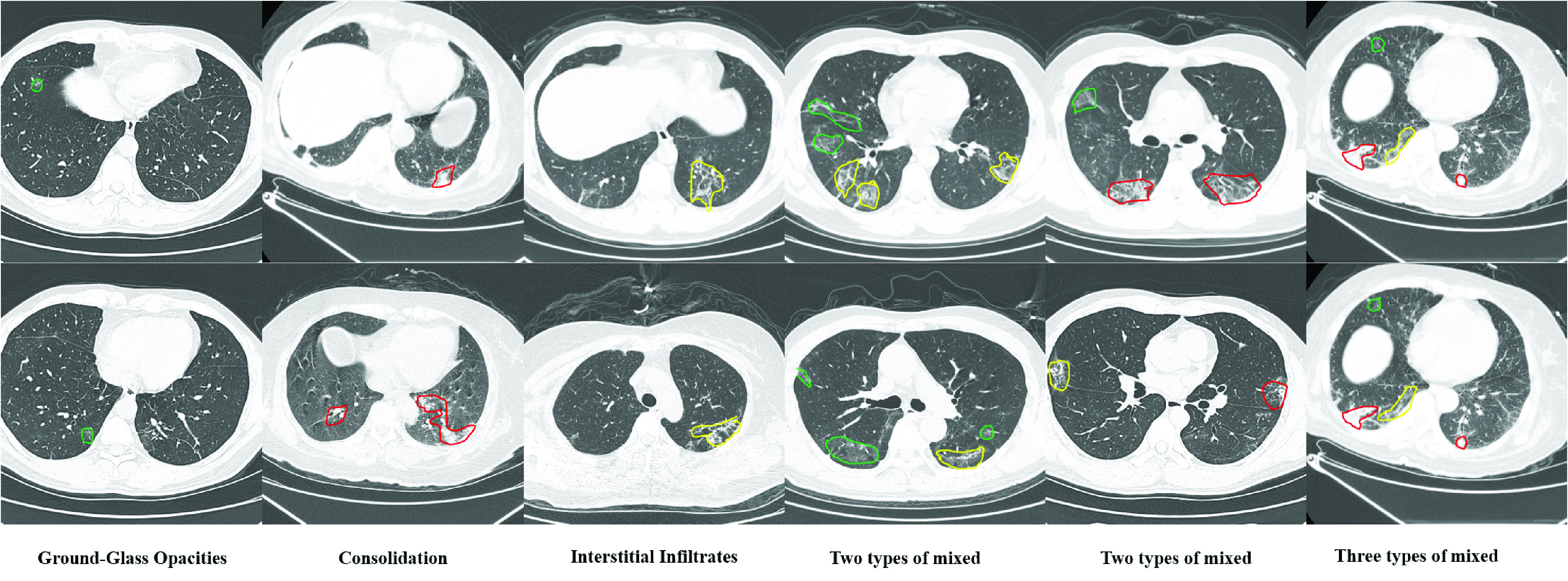
CT manifestations of different infection types. The samples in 1–3 column content single category on a CT slice. The samples in 4 and 5 columns include two categories. The last column shows the situation where the three types appear simultaneously. In the figure, green represents ground-glass opacities, yellow represents interstitial infiltrates and red represents consolidation.

The use of deep learning is gradually increasing, and is used in various applications, such as automatic driving, machine learning, face recognition, medical image processing [12]–[14]. It is an effective approach that uses deep learning for COVID-19 classification and lesion segmentation. Many significant works on COVID-19 have been proposed in [15]–[18]. For patients infected with COVID-19, different infection degree great influence on the treatment. Accurate segmentation of different types of lung infection areas can assist doctors in specific treatment. However, for many reasons, accurate segmentation of COVID-19 is a very difficult task. First, different types of infected areas have various complex appearances. For example, ground-glass opacities present diffuse bilateral pulmonary ground-glass irregular small nodules and consolidation presents irregular solid. Secondly, the size of different infection types varies greatly, as shown in Fig.1. And the same CT image may have multiple different types of infected areas.

This article designs a deep learning neural network to segment COVID-19 lesions into three categories: ground-glass opacities, interstitial infiltrates and consolidation on CT. There are many common image segmentation deep learning networks, such as FCN [19], SegNet [20], U-net [21] etc. Among them, U-net and improved network (U-Net++ [22]) are widely used in medical image segmentation. This paper proposes a novel multi-scale discriminative network (MSD-Net) for automated and accurate segmentation COVID-19 CT images. The main contribution of the network is:

1) We propose a multi-scale discriminative segmentation network (MSD-Net), which combined pyramid convolution block (PCM), channel attention block (CAB) and residual refinement block (RRB), for the accurate segmentation of COVID-19 lung infection into three categories.
2) Due to the difference in the size of the three types of lesion areas on CT, we propose the pyramid convolution block (PCB) to increase the receptive field of the network and further optimize the segmentation results.
3) A new attention module named channel attention block (CAB) is proposed to fuse two adjacent stages with attention mechanism. Experimental results show that CAB can significantly improve the segmentation effect of the network compared with the existing attention module.

The rest of this paper is organized as follows. Section 2 introduces some work related to our method. Section 3 describes the proposed method in detail and dataset. We report the experimental results in Section 4 and the limitations of the study in Section 5. At last Section 6 concludes the paper.

## II. Related Work

### A. Image Segmentation

Image semantic segmentation is one of the important fields of computer vision, which can classify images at the pixel level. And many classic segmentation networks based on deep learning have been proposed so far. Fully Convolutional Network (FCN) [19] used deep learning for semantic segmentation for the first time. It replaced fully connected layers of CNN into convolutional layers, achieving pixel-wise classification. Many subsequent segmentation networks of encoder-decoder architecture are developed from FCNs. Badrinarayanan [20] proposed SegNet on the basis of FCNs. SegNet is a typical encoder - decoder structure and the decoder uses the max-pooling indices received from the corresponding encoder to perform upsampling of the input feature map. PSPNet [23] used pyramid pooling module to fuse multi-scale context information, and our model used pyramid convolution block (PCB) to achieve multi-scale receptive fields. Ronneberger [21] proposed U-Net that was suitable for segmentation of biomedical images. Now it has been applied in many fields. U-Net++ [22] added a series of dense, nested skip connections to improve U-Net. And Attention U-Net [24] introduced an attention mechanism to achieve better segmentation. The attention gate can suppress the characteristic response of irrelevant background areas like our channel attention block (CAB). Çiçek, Ö [25] proposed 3D U-Net which converted 2D operations in U-Net to 3D. And for practical application, Paszke A [26] proposed an efficient neural network, called E-Net, which has fast running speed and high accuracy. RefineNet [27] introduced multi-resolution fusion module, residual convolution unit and chained residual pooling to achieve high resolution segmentation. Residual convolution unit is similar to our residual refinement block (RRB) which is improved from residual block. The Deeplab v1 [28] introduced dilated convolution, which can increase the receptive field. The Deeplab v2 [29] proposed atrous spatial pyramid pooling (ASPP) which used multiple filters with different rates to capture targets and context at multiple scales.

### B. Medical Image Segmentation

Milletari [30] proposed a U-net based segmentation network for prostate MRI images called V-Net. The V-Net combines different modalities of MRI images to realize the end-to-end prostate segmentation. The NVIDIA Lab’s Myronenko [31] proposed a new glioma segmentation network. The network adds a decoder to the traditional encoder-decoder structure to reconstruct the input image encoder feature extraction results. The DUNet network proposed by Jin *et al.* [32] introduces the idea of deformable convolution on the basis of U-Net. It uses the local features of retinal vessels to achieve the end-to-end segmentation task. The DUNet can adaptively adjust the size of the convolution kernel according to the thickness and shape of the segmented blood vessel, and obtain accurate segmentation results of the blood vessel based on multi-scale convolution. Fan *et al.* [33] proposed a new COVID-19 Lung Infection Segmentation Network (Inf-Net) to automatically segment infected regions. The Inf-Net uses a parallel partial decoder to generate a global map and aggregate high-level features. Then it utilizes explicit edge-attention and implicit reverse attention to enhance the representations. Moreover, the author used weak supervision to train the network. The dataset is 50 CT images with ground-truth labels and 1600 CT images with pseudo labels. The DSC of this model is 0.739, the sensitivity is 0.725, and the specificity is 0.960. Amyar *et al.* [34] created a multitask deep learning network for COVID-19 classification and segmentation. The DSC of this model is higher than 0.78. The paper used 1044 patients including 100 normal ones, 449 patients with COVID-19 and 495 of different kinds of pathology for experiments. Wang *et al.* [35] proposed an automatic segmentation network named COPLE-Net. It is characterized by its noise-robust. The DSC of the result is 80.72 ± 9.96. This paper used 558 COVID-19 patients for the experiment. Wang *et al.* [36] developed a weakly-supervised deep learning framework for COVID-19 classification and lesion localization on CT. In this paper, a 3D framework DeCoVNet is designed to predict the probability of COVID-19 infection. By combining the CAM activation region in DeCoVNet and the unsupervised connected components, COVID-19 infection areas are located. This paper used 499 CT volumes for training, and 131 CT volumes for testing. The algorithm obtained 0.976 PR AUC and 0.959 ROC AUC.

## III. Material and Methods

### A. Data Collection

The COVID-19 CT segmentation dataset provided by The Affiliated Hospital of Qingdao University contains multiple CT sequences taken on different dates from 18 COVID-19 patients and 18 without COVID-19 including 20 women and 16 men. Their age is from 23 to 67 years old. We extracted 4780 2D CT axial slices from the 3D volumes, in which 2506 slices were with COVID-19 infected lesions and 2274 without. The dataset was divided into 3824 images for training, 956 images for validation by using 5-fold cross-validation. Each image with infected lesions was equipped with multi-class labels annotated by professional doctors for identifying different lung infections (including ground-glass opacities, interstitial infiltrates, and consolidation). Table 1 lists the number of images with different infection in the dataset.

**TABLE 1:** The Number of Images With Ground-Glass Opacities, Interstitial Infiltrates, and Consolidation in the COVID-19 CT Segmentation Dataset

Infection types			Numbers	
Ground-Glass Opacities	Interstitial Infiltrates	Consolidation		
✓	✓	✓	17	
✓	✓		343	
✓		✓	33	
	✓	✓	34	2506
✓			879	
	✓		1012	
		✓	188	

Pulmonary CT infection areas of COVID-19 patients were divided into three categories: ground-glass opacities, interstitial infiltrates and consolidation. Their CT manifestations are shown in Fig.1. It can be seen in the figure that the areas of different categories vary greatly, and the size of the same category varies significantly. It is possible that multiple categories of infection areas appear in the same CT image, and the infection areas of different categories may be close to each other. These conditions increase the difficulty of the segmentation task.

### B. Network Architecture

**FIGURE 2.**
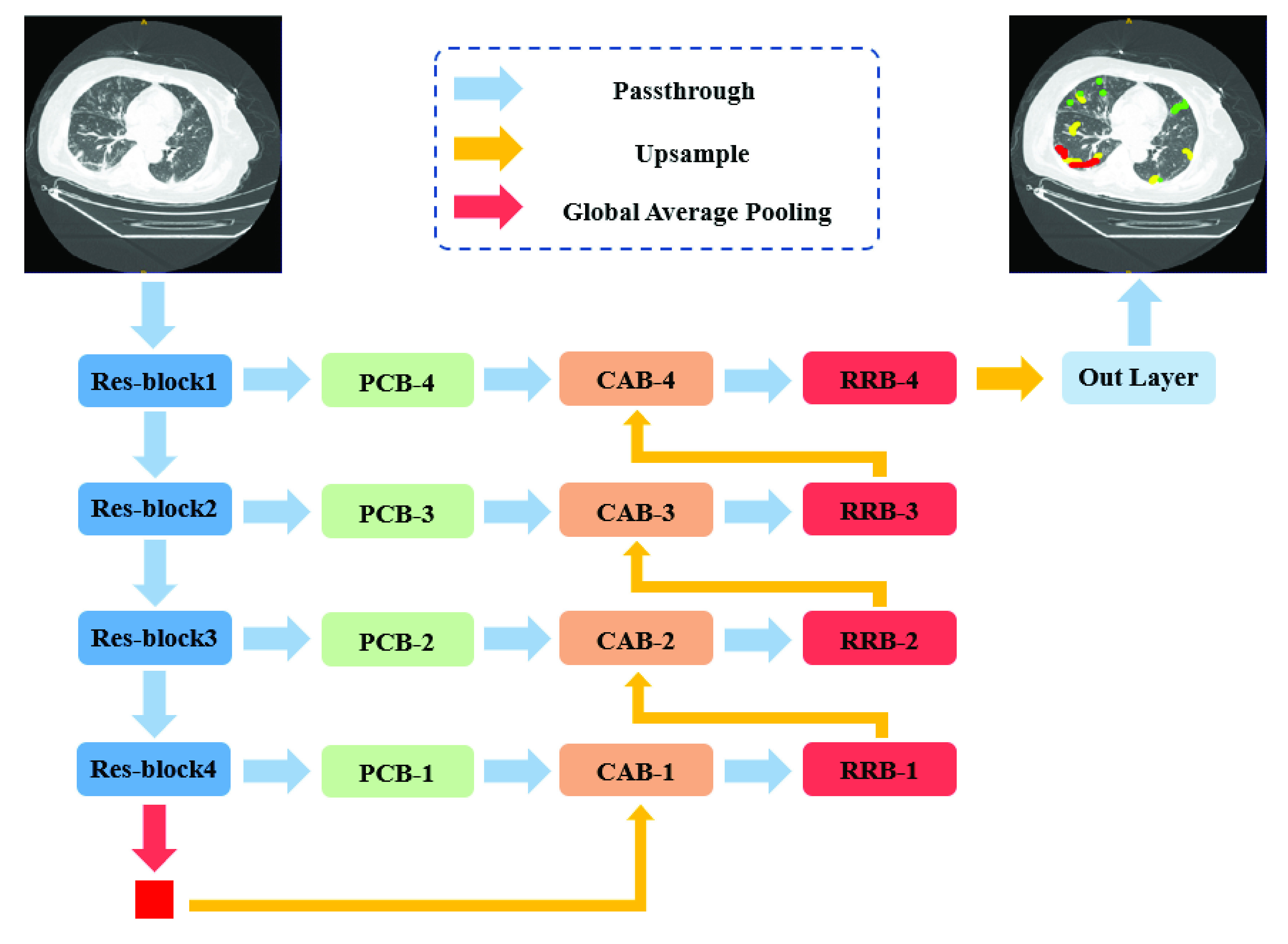
Overview of the proposed MSD-Net. PCB: Pyramid convolution block. CAB: Channel attention block. RRB: Residual refinement block. (Best viewed in color).

The U-net has often been used for medical image segmentation. In this section, we introduce our framework inspired by U-net for COVID-19 lung infection segmentation. The overall framework of the proposed multi-scale discriminative segmentation network (MSD-Net) is showed in Fig.2.

Our network takes lung CT images as input and outputs four-channel (representing ground-glass opacities, interstitial Infiltrates, consolidation, and background) segmentation results in an end-to-end manner. We used the ImageNet pre-trained ResNet-101 [37] as the backbone encoder of our model, the proposed MSD-Net has four stages of feature map scale. In addition, we added a global average pooling layer at the top of the network to extract the global semantic consistency from the encoder, because the receptive field had expanded to the full image size. The output feature maps of each stage are fed into a pyramid convolution block (PCB) to achieve multi-scale information. The PCBs effectively expand the receptive field for reducing the loss of spatial positioning information. Since the outputs of encoders have weak semantic information and the features generated by corresponding decoders have strong semantic information, we concatenate the PCB output features and the output of corresponding decoder as the inputs of the channel attention block (CAB) to obtain a channel-wise attention vector. The channel-wise attention vector contains the strong semantic information of decoder features, which can generate features with more discriminative capability. The feature maps of each stage in our network all go through the residual refinement block (RRB), which can further strengthen the discriminant ability of each stages.

### C. Pyramid Convolution Block

**FIGURE 3.**
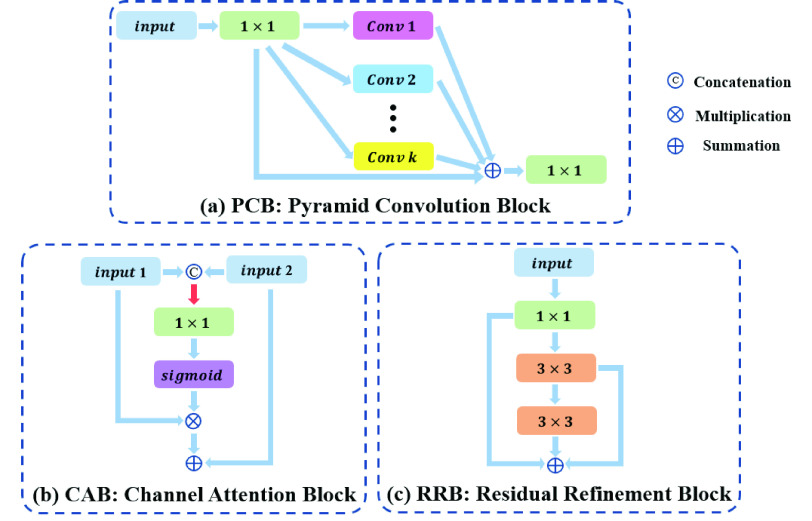
Detailed structures of the (a) pyramid convolution block, (b) channel attention block, and (c) residual refinement block.

The idea of pyramid convolution block (PCB) aims to achieve multi-scale receptive fields of input feature maps. The PCBs following four Res-block stages combine different sizes and number of convolution kernels, as illustrated in Fig.3(a). It expands receptive field for reducing the loss of spatial positioning information by using several large kernel convolutions. For each PCB-k ($k \in \{1,2,3,4\}$), there are k kernels with corresponding sizes. The corresponding size of each PCB is from $4\,\,k-1$ to $8\,\,k-5$ which is spaced by 4. For example, the kernel sizes of PCB-3 are $11 \times 11,15 \times 15,19 \times 19$ with $k=3$. In order to reduce computational complexity, we employ depth-wise separable convolutions to reduce the parameters of the pyramid convolution. The input and output channels of the PCB have not changed. The channel information of the PCB is shown in Table 2.

**TABLE 2:** The Channel Information of the Pyramid Convolution Block (PCB)

Input channels	PCB-1	PCB-2	PCB-3	PCB-4
$1 \times 1$	1024	512	256	64
Conv1	256	128	64	16
Conv2		128	64	16
Conv3			64	16
Conv4				16
$1\times 1$	256	128	64	16
Output channels	1024	512	256	64

### D. Channel Attention Block

Fig.3(b) illustrates the structure of channel attention block (CAB). The two inputs of CAB, which from two adjacent stages, are concatenated and pass through a global average pooling to generate a vector with global information. Then we use a $1\times 1$ convolution and a sigmoid activation layer to obtain the channel-wise attention vector. A channel-wise multiply operation will apply on the channel-wise attention vector and PCB output feature maps to enhance the semantic discrimination of features. Finally, the enhanced feature maps are combined with the later-features as the output of CAB. This module does not change the number of channels.

### E. Residual Refinement Block

The structure of residual refinement block is shown in Fig.3(c). Inspired by the architecture of ResNet [37], we designed the RRB based on the residual block (RB). The first component of RRB is a $1\times 1$ convolution to adjust the channel number of feature maps and the following is a basic residual block to refine the feature map. The outputs of all convolutions in RRB are then summed with the input together as the output. The feature maps output by CAB in each stage will go through the RRB. With this design, the RRB can further strengthen the discriminant ability of each stage. The channel information of the RRB is shown in Table 3.

**TABLE 3:** The Channel Information of the Residual Refinement Block (RRB)

Input channels	RRB-1	RRB-2	RRB-3	RRB-4
$1 \times 1$	1024	512	256	64
$3 \times 3$	512	256	64	32
$3 \times 3$	512	256	64	32
Output channels	512	256	64	32

### F. Learning Objectives

In general, loss function plays a significant role in training the network. Since the class imbalance of the different infection region, especially the proportion of negatives (background areas) in the whole image is too large, we use focal loss [38] to reduce the influence of class imbalance and improve the network sensitivity to the under-represented categories. The focal loss of class $c$ can be computed as:

\begin{align*}\mathrm {F L}\left ({p_{j}}\right)=-a_{j}\left ({1-p_{j}}\right)^{\Upsilon } \log \left ({p_{j}}\right), \quad j \in \{0,1,\ldots,c-1\} \\ {}\tag{1}\end{align*}

where $p_{c}$ is the predicted probability of category $c$. The symbol $\gamma $ represents the modulating factor of focal loss, which in our experiment is set to 2.0. The class weight $a_{c}$ is assigned based on the proportion of pixels in different regions. Let $N=\left \{{N_{1}, N_{2}, N_{3}, N_{4}}\right \}$ represents the set of the pixel numbers in four different regions, the class weight $a_{c}$ can be computed as:

\begin{equation*}a_{j}=\frac {\exp \left ({\frac {\min (N)}{N_{j}}}\right)}{\sum _{j} \exp \left ({\frac {\min (N)}{N_{j}}}\right)},\quad j \in \{0,1,\ldots,c-1\}\tag{2}\end{equation*}

## IV. Experiments and Results

### A. Implementation Details

The proposed method was implemented in Pytorch [39]. All the COVID-19 CT images in our experiments had been resized to $512\times 512$. In order to reduce the influence of overfitting caused by limited datasets, we employed several data augmentation operations, including random rotation and random flipping (up-down or left-right in x-y planes). The Adam optimizer [40] had been employed for training our model in an end-to-end manner with an initial learning rate of 0.001 and betas of (0.9, 0.999). The learning rate decayed by 0.1 every 100 epochs. The batch size was set to 8 on an NVIDIA GeForce GTX 1080ti GPU with 11GB memory.

Training MSD-Net on the training set which consisted of 3824 CT images took about 4 hours and testing a CT image costed an average of 0.023 seconds on an NVIDIA GeForce GTX 1080Ti GPU.

### B. Evaluation Criteria

We implemented four widely used medical image segmentation models, including U-Net [21], U-Net++ [22], and U-Net + CBAM [41] and Attention U-net [24] for a straight comparison on the COVID-19 CT segmentation dataset. These state-of-the-art models and the proposed network were both evaluated using 5-fold cross-validation. The metrics employed to quantitatively evaluate segmentation was dice similarity coefficient (DSC) [42], sensitivity and specificity. DSC is used to evaluate the similarity between the predicted segmentation result $P$ and ground truth $G$, which is calculated as:

\begin{equation*}\mathrm {DSC}(G, P)=\frac {2|G \cap P|}{|G|+|P|}\tag{3}\end{equation*}

where $|\cdot|$ represents the number of voxels. The value of DSC ranges from 0 to 1, and a larger value represents a more accurate segmentation result. Sensitivity and Specificity evaluate the segmentation from the aspect of pixel-wise classification accuracy, as shown in following:

\begin{align*}\text {Sensitivity}=&\frac {\mathrm {T P}}{\mathrm {T P+F N}}\tag{4}\\ \text {Specificity}=&\frac {\mathrm {T N}}{\mathrm {T N+F P}}\tag{5}\end{align*}

### C. Segmentation Results and Comparisons

**FIGURE 4.**
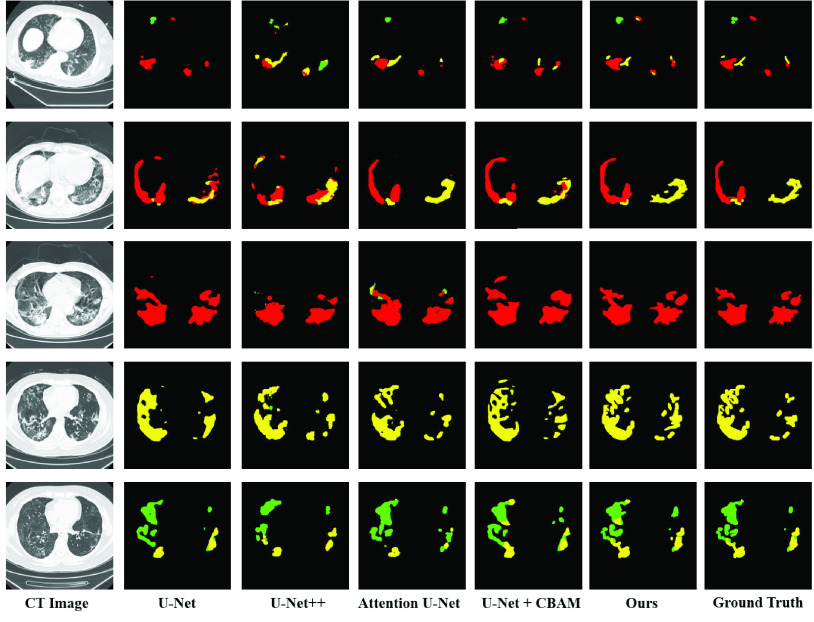
Multi-class lung infection segmentation results obtained by the proposed model and other methods. The green, yellow, and red labels indicate the ground-glass opacities, interstitial infiltrates, and consolidation, respectively. (Best viewed in color).

The multi-class segmentation results on CT images of COVID-19 are shown in Fig.4 and the statistical results are listed in Table 4. To compare the multi-class infection segmentation performance, we evaluated the proposed multi-scale discriminative segmentation network against four deep encoder-decoder networks which had been widely used for medical image segmentation. Table 4 gives the Dice similarity coefficient (DSC), Sensitivity (Sen.), and Specificity (Spec.) obtained by the models in the 5-fold cross-validation on the COVID-19 CT segmentation dataset.

**TABLE 4:** Quantitative Comparison of the Proposed Model and Four Widely Used Deep Models for Multi-Class Infection Segmentation. (Mean ± Standard Deviation of DSC, Sensitivity, and Specificity, Best Results are Highlighted in Bold)

Method	Infection type	DSC	Sen.	Spec.	P-value
U-Net [21]	Ground-Glass Opacities	0.6034 ± 0.073	0.72 31 ± 0.011	0.9775 ± 0.010	$1.09 \times 10^{-8}$
	Interstitial Infiltrates	0.6423 ± 0.081	0.7361 ± 0.025	0.9756 ± 0.008	
	Consolidation	0.7526 ± 0.036	0.8209 ± 0.026	0.9863 ± 0.005	
U-Net++ [22]	Ground-Glass Opacities	0.7160 ± 0.052	0.8017 ± 0.014	0.9675 ± 0.004	$3.15 \times 10^{-5}$
	Interstitial Infiltrates	0.6971 ± 0.034	0.7829 ± 0.018	0.9847 ± 0.008	
	Consolidation	0.8041 ± 0.042	0.8172 ± 0.009	0.9865 ± 0.003	
Attention U-Net [24]	Ground-Glass Opacities	0.7226 ± 0.026	0.8038 ± 0.019	0.9665 ± 0.012	$1.56 \times 10^{-4}$
	Interstitial Infiltrates	0.7158 ± 0.024	0.7953 ± 0.011	0.9812 ± 0.007	
	Consolidation	0.8012 ± 0.041	0.8147 ± 0.015	0.9814 ± 0.008	
U-Net+CBAM [41]	Ground-Glass Opacities	0.7037 ± 0.039	0.8172 ± 0.013	0.9675 ± 0.005	$7.22 \times 10^{-6}$
	Interstitial Infiltrates	0.6824 ± 0.032	0.7975 ± 0.018	0.9431 ± 0.008	
	Consolidation	0.8005 ± 0.052	0.8727 ± 0.021	0.9827 ± 0.004	
Our network	Ground-Glass Opacities	0.7422 ± 0.038	0.8593 ± 0.018	0.9742 ± 0.005	
	Interstitial Infiltrates	0.7384 ± 0.021	0.8268 ± 0.020	0.9869 ± 0.005	
	Consolidation	0.8769 ± 0.015	0.8645 ± 0.017	0.9889 ± 0.007

**TABLE 5:** Quantitative Evaluation Results of Different Modules Used in Our Proposed Model. RB: Residual Block, RRB: Residual Refinement Block, PCB: Pyramid Convolution Block, CAB: Channel Attention Block

Method	Infection type	DSC	Sen.	Spec.
(1) ResNet-101+RB (Backbone)	Ground-Glass Opacities	0.7034 ± 0.044	0.8011 ± 0.019	0.9714 ± 0.011
	Interstitial Infiltrates	0.6958 ± 0.026	0.7969 ± 0.020	0.9799 ± 0.009
	Consolidation	0.7934 ± 0.020	0.8289 ± 0.025	0.9806 ± 0.012
(2) ResNet-101+RRB	Ground-Glass Opacities	0.7148 ± 0.041	0.8246 ± 0.021	0.9729 ± 0.007
	Interstitial Infiltrates	0.7198 ± 0.021	0.8101 ± 0.018	0.9832 ± 0.010
	Consolidation	0.8066 ± 0.020	0.8536 ± 0.018	0.9833 ± 0.009
(3) ResNet-101+RRB+CAB	Ground-Glass Opacities	0.7278 ± 0.039	0.8402 ± 0.019	0.9781 ± 0.006
	Interstitial Infiltrates	0.7293 ± 0.026	0.8145 ± 0.0201	0.9862 ± 0.008
	Consolidation	0.8605 ± 0.0151	0.8502 ± 0.019	0.9891 ± 0.005
(4) ResNet-101+RRB+CAB+PCB	Ground-Glass Opacities	0.7422 ± 0.038	0.8593 ± 0.018	0.9742 ± 0.005
	Interstitial Infiltrates	0.7384 ± 0.021	0.8268 ± 0.020	0.9869 ± 0.005
	Consolidation	0.8769 ± 0.015	0.8645 ± 0.017	0.9889 ± 0.007

It can be observed that the baseline U-Net achieves much lower DSC and sensitivity for all categories. The Attention U-Net [24] performed better on DSC and the U-Net + CBAM [41] performed better on sensitivity than U-Net and U-Net++ because of involving the attention modules. Compared with Attention U-Net [24], our MSD-Net improved the average DSC from 72.26% to 74.22% for ground-glass opacities, 71.58% to 73.84% for interstitial infiltrates and 80.12% to 87.69% for consolidation respectively. The MSD-Net also achieved significant improvement on sensitivity. Compared with U-Net + CBAM [41], our model effectively improved more than 4 percentage of sensitivity from 81.72% to 85.93% for ground-glass opacities. And for interstitial infiltrates, the sensitivity increased from 79.75% to 82.68%. It can be seen that the proposed model had better segmentation performance on ground-glass opacities and interstitial infiltrates categories, which was also the difficulty of COVID-19 accurate segmentation. We attributed the improvement of our MSD-Net to the pyramid convolution block and channel attention, which provided significant multi-scale discriminant feature maps. The specificity is much higher than sensitivity and varied slightly for different methods because that the true negative pixels without COVID-19 were far more than the true positive samples. All the p values are also given in Table 4. It is obviously that our proposed model was significantly different from other models with p<0.05.

Fig.4 shows the qualitative comparisons of our model with the other four medical segmentation networks. It can be observed that the infection lesions of the three categories were with rich diversity in shape and area. This attribute caused the difficulties of lesion segmentation. Our proposed pyramid multi-scale and attention model can solve the problem to some extent. The comparison results intuitively illustrated that our MSD-Net performed better than other start-of-the-art segmentation models. Both the qualitative and quantitative comparisons proved that our method can produced more accurate segmentation results that were close to the ground truth with less mis-segmented tissue. The PCB and CAB can effectively improve the ability to segment all categories, especially the high sensitivity to ground-glass opacitie and interstitial infiltrates.

### D. Ablation Experiments

We conducted several ablation experiments to evaluate the contributions of each key component to the overall performance of our proposed model, including pyramid convolution block (PCB), channel attention block (CAB), and residual refinement block (RRB). The results obtained by applying the proposed network with RB (residual block), RRB, CAB, and PCB to the COVID-19 CT segmentation dataset are given in Table 3. It shows that using RRB to replace RB slightly increases the performance at both three infection regions (comparing the results in Table3 (1) and (2)). Employing CAB greatly improves the segmentation performance at consolidation areas (comparing the results of (2) and (3)), which suggests that CAB can enable our model to generate more discriminative and accurate results. The PCB module effectively increases the performance (comparing the results of (3) and (4)) in terms of all metrics. This suggests that introducing the PCB component can enable our model to acquire multi-scale discriminative information and distinguish true infected areas accurately.

### E. Visualization Studies

**FIGURE 5.**
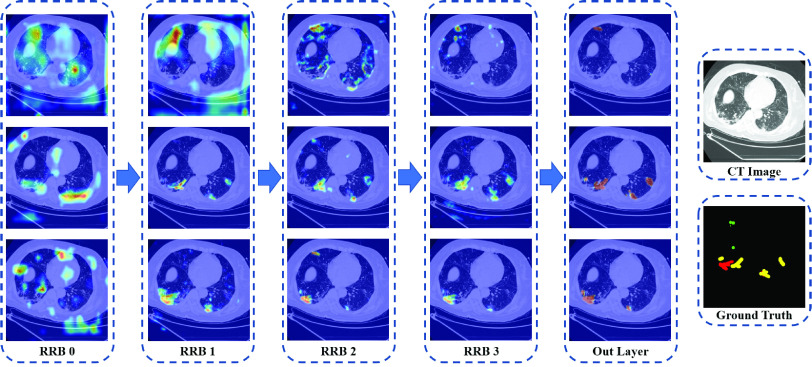
Examples of three categories feature maps generated by each decoder-layer, the hotter color represents the higher response value. RRB: residual refinement block. (Best viewed in color).

The proposed MSD-Net can achieve more discriminative and accurate results by using the semantic information. In order to qualitatively illustrate the effectiveness of the network, we selected several feature maps from each decoder layer. The visualization of the feature maps provided some insight view of the network segmentation performance. As shown in Fig.5, the hotter color represents the higher response value. Each column is the output feature maps from the decoder RRBs block, and each raw indicate the high response areas of the three infection lesion categories. It can be observed that our designed network can indicate how much attention should be paid to the infection areas.

### F. Robustness

**FIGURE 6.**
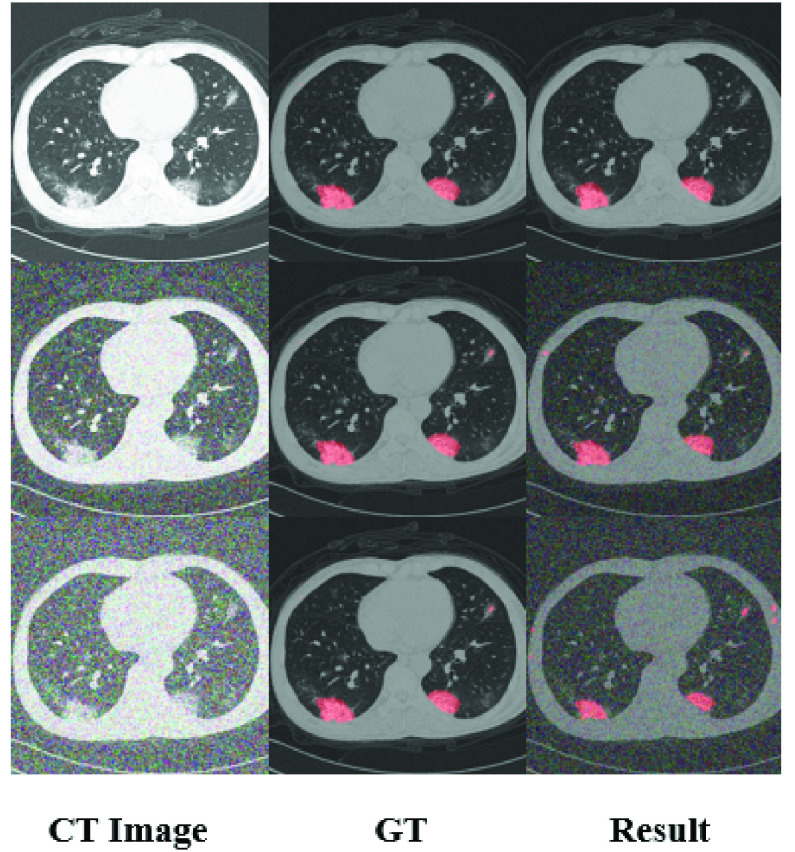
Results with different noise. The first column is the input CT images. The second column is the ground truth, and the third column is the segmentation results. The first row is the original input, the second row is the input which is infected by gaussian noise with a variance of 0.05. The third row is infected with a variance of 0.10.

When the input CT data are infected with a higher level (but still reasonable amount) of noise and artifacts, the performance of the proposed model is still satisfied. We add different types of noise to the input(gaussian noise with variances of 0.05 and 0.10), and the results show that our network has achieved good segmentation results, as shown in Fig. 6.

## V. Limitations of the Study

Our study has some limitations.In terms of insufficient number of samples, there are fewer samples of mild symptoms, and most of the patients are moderate and severe patients. Mild symptoms are vague and difficult to distinguish. We use Focal Loss [38] to solve the problem to a certain extent. To further optimize the overall network, a strong backbone model and optimal loss functions can be designed in the future. Also new data enhancement methods can be concerned to improve the accuracy.

## VI. Conclusion

Because the spread of COVID-19 has not been brought under control and testing kits are in short supply, the use of deep learning for auxiliary diagnosis of COVID-19 is of great significance. In this paper, we proposed the multi-scale discriminative segmentation network (MSD-Net) which can perform multi-class infection segmentation. The CT infection categories are ground-glass opacities, interstitial infiltrates and consolidation according to lesions degree of symptoms and labeled by professional doctors. We involved the pyramid convolution block and design an attention block in the proposed MSD-Net to effectively increase the segmentation results. Compared with other state-of-the-art segmented networks, our method achieved significant performance. The DSC indicators of the three infection categories were 0.7422,0.7384,0.8769 respectively. And for sensitivity and specificity, the results were (0.8593, 0.9742), (0.8268,0.9869), (0.8645,0.9889). Experimental results show that the proposed MSD-Net can effectively segment CT infection lesions for COVID-19. The network also provides a quantitative auxiliary analysis method for the diagnosis of COVID-19.

## References

[1] J. F.-W. Chan, “A familial cluster of pneumonia associated with the 2019 novel coronavirus indicating person-to-person transmission: A study of a family cluster,” Lancet, vol. 395, no. 10223, pp. 514–523, Feb. 2020. 31986261 10.1016/S0140-6736(20)30154-9 PMC7159286

[2] S. Yang, L. Jiang, Z. Cao, L. Wang, J. Cao, R. Feng, Z. Zhang, X. Xue, Y. Shi, and F. Shan, “Deep learning for detecting corona virus disease 2019 (COVID-19) on high-resolution computed tomography: A pilot study,” Ann. Transl. Med., vol. 8, no. 7, p. 450, Apr. 2020. 32395494 10.21037/atm.2020.03.132 PMC7210135

[3] N. Zhu, D. Zhang, W. Wang, X. Li, B. Yang, J. Song, X. Zhao, and B. Huang, W. Shi, R. Lu, and P. Niu, “A novel coronavirus from patients with pneumonia in China, 2019,” New England J. Med., vol. 382, no. 8, pp. 727–733, 2020. 31978945 10.1056/NEJMoa2001017 PMC7092803

[4] World Health Organization. (2020). Who Director-General’s Opening Remarks at the Media Briefing on Covid-19-11 March 2020. [Online]. Available: https://www.who.int/dg/speeches/detail/who-director-generals-opening-remarks-at-the-media-briefing-on-covid-19-11-march-2020

[5] E. Dong, H. Du, and L. Gardner, “An interactive Web-based dashboard to track COVID-19 in real time,” Lancet Infectious Diseases, vol. 20, no. 5, pp. 533–534, 5 2020. 32087114 10.1016/S1473-3099(20)30120-1 PMC7159018

[6] WHO Clinical Management of COVID-19. [Online]. Available: https://www.who.int/publications/i/item/clinical-management-of-severeacute-respiratory-infection-when-novel-coronavirus-(ncov)-infection-issuspected

[7] C. Huang, Y. Wang, X. Li, L. Ren, J. Zhao, Y. Hu, L. Zhang, G. Fan, J. Xu, X. Gu, and Z. Cheng, “Clinical features of patients infected with 2019 novel coronavirus in Wuhan, China,” Lancet, vol. 395, no. 10223, pp. 497–506, 2020. 31986264 10.1016/S0140-6736(20)30183-5 PMC7159299

[8] J. Lei, J. Li, X. Li, and X. Qi, “CT Imaging of the 2019 Novel Coronavirus (2019-nCoV) Pneumonia,” Radiology, vol. 295, no. 1, p. 18, 2020. 32003646 10.1148/radiol.2020200236 PMC7194019

[9] Y.-H. Xu, J.-H. Dong, W.-M. An, X.-Y. Lv, X.-P. Yin, J.-Z. Zhang, L. Dong, X. Ma, H.-J. Zhang, and B.-L. Gao, “Clinical and computed tomographic imaging features of novel coronavirus pneumonia caused by SARS-CoV-2,” J. Infection, vol. 80, no. 4, pp. 394–400, Apr. 2020. 10.1016/j.jinf.2020.02.017 PMC7102535 32109443

[10] F. Shan, Y. Gao, J. Wang, W. Shi, N. Shi, M. Han, Z. Xue, D. Shen, and Y. Shi, “Lung infection quantification of COVID-19 in CT images with deep learning,” 2020, arXiv:2003.04655. [Online]. Available: http://arxiv.org/abs/2003.04655

[11] A. A. A. Setio, F. Ciompi, G. Litjens, P. Gerke, C. Jacobs, S. J. van Riel, M. M. W. Wille, M. Naqibullah, C. I. Sanchez, and B. van Ginneken, “Pulmonary nodule detection in CT images: False positive reduction using multi-view convolutional networks,” IEEE Trans. Med. Imag., vol. 35, no. 5, pp. 1160–1169, 5 2016. 10.1109/TMI.2016.2536809 26955024

[12] X. Qin, “Transfer learning with edge attention for prostate MRI segmentation,” 2019, arXiv:1912.09847. [Online]. Available: http://arxiv.org/abs/1912.09847

[13] J. Zheng, D. Yang, Y. Zhu, W. Gu, B. Zheng, C. Bai, L. Zhao, H. Shi, J. Hu, S. Lu, W. Shi, and N. Wang, “Pulmonary nodule risk classification in adenocarcinoma from CT images using deep CNN with scale transfer module,” IET Image Process., vol. 14, no. 8, pp. 1481–1489, Jun. 2020, doi: 10.1049/iet-ipr.2019.0248.

[14] H. Kang, L. Xia, F. Yan, Z. Wan, F. Shi, H. Yuan, H. Jiang, D. Wu, H. Sui, C. Zhang, and D. Shen, “Diagnosis of coronavirus disease 2019 (COVID-19) with structured latent multi-view representation learning,” IEEE Trans. Med. Imag., vol. 39, no. 8, pp. 2606–2614, Aug. 2020, doi: 10.1109/TMI.2020.2992546. 32386147

[15] A. Waheed, M. Goyal, D. Gupta, A. Khanna, F. Al-Turjman, and P. Rogerio Pinheiro, “CovidGAN: Data augmentation using auxiliary classifier GAN for improved covid-19 detection,” IEEE Access, vol. 8, pp. 91916–91923, 2020, doi: 10.1109/ACCESS.2020.2994762. 34192100 PMC8043420

[16] H. Jelodar, Y. Wang, R. Orji, and H. Huang, “Deep sentiment classification and topic discovery on novel coronavirus or COVID-19 online discussions: NLP using LSTM recurrent neural network approach,” IEEE J. Biomed. Health Informat., early access, Jun. 9, 2020, doi: 10.1109/JBHI.2020.3001216. 32750931

[17] S. Rajaraman, J. Siegelman, P. O. Alderson, L. S. Folio, L. R. Folio, and S. K. Antani, “Iteratively pruned deep learning ensembles for COVID-19 detection in chest X-Rays,” IEEE Access, vol. 8, pp. 115041–115050, 2020, doi: 10.1109/ACCESS.2020.3003810. 32742893 PMC7394290

[18] S. Wang, B. Kang, J. Ma, X. Zeng, M. Xiao, J. Guo, M. Cai, J. Yang, Y. Li, X. Meng, and B. Xu, “A deep Learning algorithm using CT images to screen for corona virus disease (COVID-19),” MedRxiv, to be published, doi: 10.1101/2020.02.14.20023028. PMC7904034 33629156

[19] J. Long, E. Shelhamer, and T. Darrell, “Fully convolutional networks for semantic segmentation,” in Proc. IEEE Conf. Comput. Vis. Pattern Recognit. (CVPR), Jun. 2015, pp. 3431–3440. 10.1109/TPAMI.2016.2572683 27244717

[20] V. Badrinarayanan, A. Kendall, and R. Cipolla, “SegNet: A deep convolutional encoder-decoder architecture for image segmentation,” IEEE Trans. Pattern Anal. Mach. Intell., vol. 39, no. 12, pp. 2481–2495, Dec. 2017. 28060704 10.1109/TPAMI.2016.2644615

[21] O. Ronneberger, P. Fischer, and T. Brox, “U-Net: Convolutional networks for biomedical image segmentation,” in Proc. Int. Conf. Med. Image Comput. Comput.-Assist. Intervent. Springer, 2015, pp. 234–241.

[22] Z. Zhou, M. M. R. Siddiquee, N. Tajbakhsh, and J. Liang, “UNet++: A nested U-Net architecture for medical image segmentation,” in Deep Learning in Medical Image Analysis and Multimodal Learning for Clinical Decision Support. Springer, 2018, pp. 3–11. 10.1007/978-3-030-00889-5_1 PMC7329239 32613207

[23] H. Zhao, J. Shi, X. Qi, X. Wang, and J. Jia, “Pyramid scene parsing network,” in Proc. IEEE Conf. Comput. Vis. Pattern Recognit. (CVPR), Jul. 2017, pp. 2881–2890.

[24] O. Oktay, J. Schlemper, L. Le Folgoc, M. Lee, M. Heinrich, K. Misawa, K. Mori, S. McDonagh, N. Y Hammerla, B. Kainz, B. Glocker, and D. Rueckert, “Attention U-Net: Learning where to look for the pancreas,” 2018, arXiv:1804.03999. [Online]. Available: http://arxiv.org/abs/1804.03999

[25] O. Çiçek, A. Abdulkadir, S. S. Lienkamp, T. Brox, and O. Ronneberger, “3D U-Net: Learning dense, volumetric segmentation from sparse annotation,” in Proc. Int. Conf. Med. image Comput. Comput.-Assist. Intervent. Springer, 2016, pp. 424–432.

[26] A. Paszke, A. Chaurasia, S. Kim, and E. Culurciello, “ENet: A deep neural network architecture for real-time semantic segmentation,” 2016, arXiv:1606.02147. [Online]. Available: http://arxiv.org/abs/1606.02147

[27] G. Lin, A. Milan, C. Shen, and I. Reid, “RefineNet: Multi-path refinement networks for high-resolution semantic segmentation,” in Proc. IEEE Conf. Comput. Vis. Pattern Recognit. (CVPR), Jul. 2017, pp. 1925–1934.

[28] L.-C. Chen, G. Papandreou, I. Kokkinos, K. Murphy, and A. L. Yuille, “Semantic image segmentation with deep convolutional nets and fully connected CRFs,” 2014, arXiv:1412.7062. [Online]. Available: http://arxiv.org/abs/1412.7062 10.1109/TPAMI.2017.2699184 28463186

[29] L.-C. Chen, G. Papandreou, I. Kokkinos, K. Murphy, and A. L. Yuille, “DeepLab: Semantic image segmentation with deep convolutional nets, atrous convolution, and fully connected CRFs,” IEEE Trans. Pattern Anal. Mach. Intell., vol. 40, no. 4, pp. 834–848, Apr. 2018. 28463186 10.1109/TPAMI.2017.2699184

[30] F. Milletari, N. Navab, and S.-A. Ahmadi, “V-Net: Fully convolutional neural networks for volumetric medical image segmentation,” in Proc. 4th Int. Conf. 3D Vis. (3DV), Oct. 2016, pp. 565–571.

[31] A. Myronenko, “3D MRI brain tumor segmentation using autoencoder regularization,” in Proc. Int. MICCAI Brainlesion Workshop. Springer, 2018, pp. 311–320.

[32] Q. Jin, Z. Meng, T. D. Pham, Q. Chen, L. Wei, and R. Su, “DUNet: A deformable network for retinal vessel segmentation,” Knowl.-Based Syst., vol. 178, pp. 149–162, Aug. 2019.

[33] D.-P. Fan, T. Zhou, G.-P. Ji, Y. Zhou, G. Chen, H. Fu, J. Shen, and L. Shao, “Inf-Net: Automatic COVID-19 lung infection segmentation from CT images,” IEEE Trans. Med. Imag., vol. 39, no. 8, pp. 2626–2637, Aug. 2020. 10.1109/TMI.2020.2996645 32730213

[34] A. Amyar, R. Modzelewski, and S. Ruan, “Multi-task deep learning based CT imaging analysis for COVID-19: Classification and Segmentation,” MedRxiv, to be published, doi: 10.1101/2020.04.16.20064709. PMC7543793 33065387

[35] G. Wang, X. Liu, C. Li, Z. Xu, J. Ruan, H. Zhu, T. Meng, K. Li, N. Huang, and S. Zhang, “A noise-robust framework for automatic segmentation of COVID-19 pneumonia lesions from CT images,” IEEE Trans. Med. Imag., vol. 39, no. 8, pp. 2653–2663, Aug. 2020, doi: 10.1109/TMI.2020.3000314. PMC8544954 32730215

[36] X. Wang, X. Deng, Q. Fu, Q. Zhou, J. Feng, H. Ma, W. Liu, and C. Zheng, “A weakly-supervised framework for COVID-19 classification and lesion localization from chest CT,” IEEE Trans. Med. Imag., vol. 39, no. 8, pp. 2615–2625, Aug. 2020, doi: 10.1109/TMI.2020.2995965. 33156775

[37] K. He, X. Zhang, S. Ren, and J. Sun, “Deep residual learning for image recognition,” in Proc. IEEE Conf. Comput. Vis. Pattern Recognit. (CVPR), Jun. 2016, pp. 770–778.

[38] T.-Y. Lin, P. Goyal, R. Girshick, K. He, and P. Dollar, “Focal loss for dense object detection,” in Proc. IEEE Int. Conf. Comput. Vis. (ICCV), Oct. 2017, pp. 2980–2988. 10.1109/TPAMI.2018.2858826 30040631

[39] A. Paszke, S. Gross, F. Massa, A. Lerer, J. Bradbury, G. Chanan, T. Killeen, Z. Lin, N. Gimelshein, L. Antiga, and A. Desmaison, “Pytorch: An imperative style, high-performance deep learning library,” in Proc. Adv. Neural Inf. Process. Syst., vol. 2019, pp. 8026–8037.

[40] K. Da, “A method for stochastic optimization,” 2014, arXiv:1412.6980. [Online]. Available: https://arxiv.org/abs/1412.6980

[41] S. Woo, J. Park, J.-Y. Lee, and I. So Kweon, “CBAM: Convolutional block attention module,” in Proc. Eur. Conf. Comput. Vis. (ECCV), Sep. 2018, pp. 3–19.

[42] G. Litjens, “Evaluation of prostate segmentation algorithms for MRI: The PROMISE12 challenge,” Med. Image Anal., vol. 18, no. 2, pp. 359–373, Feb. 2014. 24418598 10.1016/j.media.2013.12.002 PMC4137968

